# Detection of SARS-CoV-2 Viral Genome and Viral Nucleocapsid in Various Organs and Systems

**DOI:** 10.3390/ijms25115755

**Published:** 2024-05-25

**Authors:** George Călin Oprinca, Cosmin-Ioan Mohor, Alina-Simona Bereanu, Lilioara-Alexandra Oprinca-Muja, Iancu Bogdan-Duică, Sorin Radu Fleacă, Adrian Hașegan, Atasie Diter, Ioana Boeraș, Adrian Nicolae Cristian, Elena-Teodora Tâlvan, Călin Ilie Mohor

**Affiliations:** 1 Faculty of Medicine, Lucian Blaga University of Sibiu, 550169 Sibiu, Romania; georgecalin.oprinca@ulbsibiu.ro (G.C.O.); lilioaraalexandra.muja@ulbsibiu.ro (L.-A.O.-M.); radu.fleaca@ulbsibiu.ro (S.R.F.); adrian.hasegan@ulbsibiu.ro (A.H.); atasie.diter@ulbsibiu.ro (A.D.); adrian.cristian@ulbsibiu.ro (A.N.C.); elena.talvan@ulbsibiu.ro (E.-T.T.); calin.mohor@ulbsibiu.ro (C.I.M.); 2 County Clinical Emergency Hospital, Bld. Corneliu Coposu, Nr. 2-4, 550245 Sibiu, Romania; 3 Faculty of Sciences, Lucian Blaga University of Sibiu, 550012 Sibiu, Romania; ioana.boeras@ulbsibiu.ro

**Keywords:** SARS-CoV-2, autopsy, histopathology, immunohistochemistry, molecular, heart, kidney, liver, spleen, intestines

## Abstract

While considerable attention has been devoted to respiratory manifestations, such as pneumonia and acute respiratory distress syndrome (ARDS), emerging evidence underlines the significance of extrapulmonary involvement. In this study, we examined 15 hospitalized patients who succumbed to severe complications following SARS-CoV-2 infection. These patients were admitted to the Sibiu County Clinical Emergency Hospital in Sibiu, Romania, between March and October 2021. All patients were ethnic Romanians. Conducted within a COVID-19-restricted environment and adhering to national safety protocols, autopsies provided a comprehensive understanding of the disease’s multisystemic impact. Detailed macroscopic evaluations and histopathological analyses of myocardial, renal, hepatic, splenic, and gastrointestinal tissues were performed. Additionally, reverse transcription-quantitative polymerase chain reaction (rt-qPCR) assays and immunohistochemical staining were employed to detect the viral genome and nucleocapsid within the tissues. Myocardial lesions, including ischemic microstructural changes and inflammatory infiltrates, were prevalent, indicative of COVID-19’s cardiac implications, while renal pathology revealed the chronic alterations, acute tubular necrosis, and inflammatory infiltrates most evident. Hepatic examination identified hepatocellular necroinflammatory changes and hepatocytic cytopathy, highlighting the hepatic involvement of SARS-CoV-2 infection. Splenic parenchymal disorganization was prominent, indicating systemic immune dysregulation. Furthermore, gastrointestinal examinations unveiled nonspecific changes. Molecular analyses detected viral genes in various organs, with immunohistochemical assays confirming viral presence predominantly in macrophages and fibroblasts. These findings highlighted the systemic nature of SARS-CoV-2 infection, emphasizing the need for comprehensive clinical management strategies and targeted therapeutic approaches beyond respiratory systems.

## 1. Introduction

The emergence of the novel coronavirus SARS-CoV-2 in late 2019 sparked a global pandemic, challenging healthcare systems worldwide and reshaping societal norms. As the virus rapidly spread, it became increasingly evident that its impact extended beyond the respiratory system, affecting multiple organs and systems throughout the body. Understanding the microscopic alterations induced by SARS-CoV-2 infection across diverse organs is crucial for elucidating its pathogenesis, refining diagnostic approaches, and guiding therapeutic interventions. At the peak of the pandemic, clinicians and researchers were tirelessly investigating the intricate pathophysiological mechanisms underlying COVID-19.

SARS-CoV-2 is transmitted naturally through droplets from an infected host to direct, indirect, or close contacts. The virus targets epithelial cells in the nasal, oral, or conjunctival mucosa, entering and triggering viral replication [1]. This cycle involves the following several stages: the virus adheres to the host cell membrane, enters via endocytosis or membrane fusion, disassembles in the cytoplasm to release its genome, undergoes sub-genomic transcription, translates structural proteins, assembles new virions, and releases them through exocytosis to infect new cells [2]. The virus uses the spike protein on its envelope, structured as a trimer with S1 receptor-binding heads and an S2 fusion stalk. The S1 heads bind specifically to angiotensin conversion enzyme 2 (ACE2) receptors on host cells, switching positions to evade the immune system. After binding, the spike protein undergoes conformational changes mediated by cell surface proteases like transmembrane serine protease 2 (TMPRSS2) and lysosomal proteases such as cathepsins, which are necessary for fusion. Furin preactivation increases invasion efficiency in ACE2-presenting cells [3]. The virus can also enter through pinocytosis or by inducing clathrin coat formation. Inside the cell, it is transported via the vesicular system, processed from early to mature endosomes, and transported to lysosomes for degradation. When the viral envelope fuses with the lysosomal membrane, the genome is released into the cytosol for the next cycle stage [4]. The release of the viral genome initiates a complex gene expression program. First, RNA sequences translate to produce non-structural proteins within *open reading frame* (*ORF*) *1a* and *ORF1b*. The pp1ab (polyprotein 1ab) protein results from a ribosomal frameshift between *ORF1a* and *ORF1b*. With non-structural protein (nsp) 3 and nsp5 proteases’ help, 16 non-structural proteins are released, forming the replication and transcription complex (RTC). RNA synthesis is mediated by RdRp (RNA-dependent RNA polymerase)—nsp12—with cofactors nsp7 and nsp8. After RNA translation, it replicates and is included in new virions. Genomic replication starts with the transcription of a negative-sense RNA, serving as a template for positive-sense RNA production and generating more nsps and RTCs. *Transcription regulatory sequences* (*TRS*) *L* signal subgenomic mRNA transcription. Once produced, nsp16 and nsp10 methylate the 5′ end of viral mRNAs [1]. The nucleocapsid (N) protein binds to the newly formed viral RNA to package it into a virion. The N protein, with RNA-binding and dimerization domains, forms condensed structures with stress granule proteins (G3BP1). The membrane (M) protein induces N phase separation, forming structures with N, M, and RNA. This assembly occurs in the rough endoplasmic reticulum (RER) [5]. The viral genome then moves from the RER to the Golgi apparatus, where it is encapsulated in exocytic vacuoles or lysosomes, which fuse with the cytoplasmic membrane and expel the virion outside the cell, completing the replication cycle [6]. While respiratory manifestations, such as pneumonia and acute respiratory distress syndrome (ARDS), have been extensively studied, there is growing recognition of extrapulmonary involvement, ranging from cardiovascular complications to renal dysfunction and gastrointestinal manifestations. In this context, our research comprehensively explores the microscopic alterations induced by SARS-CoV-2 infection across various organs and systems.

Through meticulous histopathological examinations, immunohistochemical profiling, and the molecular analysis of postmortem tissue samples, we aim to describe the spectrum of pathological changes, ranging from acute cellular injury to chronic fibrotic changes, observed in organs such as the heart, kidneys, liver, spleen, and intestines. By shedding light on these microscopic alterations, we can elucidate the underlying pathophysiological mechanisms driving organ-specific injury in COVID-19 and also identify therapeutic targets that may aid in the early management of COVID-19-related complications.

Our research points out the importance of investigating microscopic alterations in various organs and systems in SARS-CoV-2 infection. By unraveling the intricate interplay between the virus and host tissues, we aim to contribute to the collective efforts aimed at understanding this pathogenic agent that drove the globe through a pandemic.

## 2. Results

**Figure 1:**
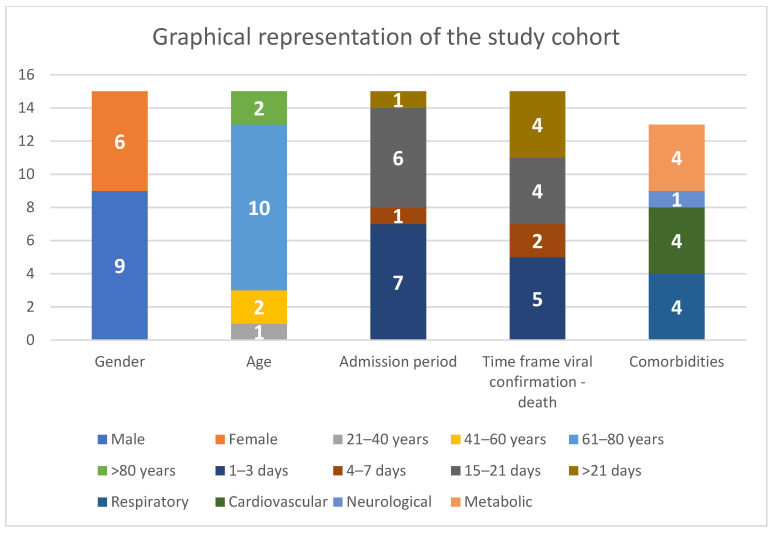
Graphical representation of the study cohort.

The study cohort comprised 9 males and 6 females, aged between 31 and 82 years, with a mean age of 67 years. All patients were hospitalized before their demise and were confirmed to have SARS-CoV-2 infection via nasopharyngeal swab testing. The duration of hospitalization ranged from 1 to 39 days, with two patients experiencing multiple admissions; only the final admission was considered for this study. The average hospital stay was 11 days. Among the 15 patients, 5 were confirmed with infection 1 to 3 days before death, while 2 died 4 to 7 days after confirmation. Additionally, 4 patients exhibited symptoms and tested positive between days 15 and 21 before death, and 4 patients died 21 days after polymerase chain reaction (PCR) confirmation, with the longest interval being 68 days. The average duration between infection confirmation and death was 15 days. Regarding comorbidities, four cases presented respiratory conditions, including chronic obstructive pulmonary disease (2 cases), pulmonary emphysema (1 case), or stage IV pulmonary neoplasm (1 case). Four patients had cardiovascular comorbidities, such as atherosclerotic disease and arterial hypertension, while one patient had neurological conditions like Parkinson’s and dementia. Additionally, four cases exhibited metabolic comorbidities, including type II diabetes (2 cases) and obesity (2 cases). Finally, three patients either had no chronic diseases or remained undiagnosed until death (Figure 1). Among the 15 patients who succumbed to confirmed SARS-CoV-2 infection, histopathological, immunohistochemical, and rt-qPCR analyses revealed that in 9 cases, the direct cause of death was acute respiratory distress syndrome induced by viral pneumonia, with four of them also showing chronic pulmonary lesions, characterized by pronounced pulmonary fibrosis alongside viral persistence. In three cases, tissue analysis indicated abnormalities consistent with COVID-19 viral pneumonia, with alterations suggestive of bacterial superinfection shortly before death. Bacterial blood cultures confirmed this diagnosis before death, and microscopic lung images showed classic bacterial pneumonia superimposed on alterations consistent with viral infection. The most common bacterial pathogens found were *Staphylococcus aureus* and *Klebsiella pneumoniae*. One 68-year-old female patient succumbed to spontaneous retroperitoneal hemorrhage following anticoagulation therapy, with microscopic and immunohistochemical findings indicative of viral pneumonia and pulmonary fibrosis. The youngest patient, a 31-year-old male, passed away after 68 days from confirmed nasopharyngeal swab testing and 39 days of hospitalization due to severe sepsis secondary to multiple bacterial infections of the respiratory tract and superinfected decubitus ulcers with multi-drug-resistant bacteria. The final case, a 71-year-old female, died from massive pleural effusion associated with stage IV pulmonary neoplasm, yet histopathological and immunohistochemical (IHC) analyses revealed changes consistent with SARS-CoV-2 pulmonary involvement.

### 2.1. Lung Tissue Analysis

Our previously published research offers a comprehensive analysis of lung tissue specimens. The histopathological spectrum of SARS-CoV-2-induced lung damage encompasses various degrees of diffuse alveolar damage characterized by exudative, proliferative, or fibrotic phases. Microscopic alterations were categorized into acute inflammatory lesions, acute alveolar lesions, vascular lesions, hemodynamic injuries, and interstitial and aberrant regenerative alterations. Acute inflammatory lesions were characterized by lympho-monocytic inflammatory infiltrates in 10 cases, polymorphonuclear (mostly neutrophils) inflammatory infiltrates in 9 cases, and mixed inflammatory infiltrates in 4 cases, often accompanied by the presence of macrophages in the alveolar space in 9 cases. Acute alveolar lesions were represented by hyaline membrane formation in 9 cases, type II pneumocyte hyperplasia in 13 cases, pneumocytes with cytopathic effects in 10 cases, and multinucleated giant cell-like pneumocyte aggregates in 6 cases. Hemodynamic injuries observed in our study cohort included pulmonary edema in 7 cases and diffuse alveolar hemorrhage in 8 cases, while vascular lesions were characterized by lymphocytic vasculitic reactions in 3 patients and microvasculature thrombosis in 5 patients. Interstitial and aberrant regenerative alterations consisted of organizing pneumonia in 4 cases, interstitial fibrosis in 8 cases, and squamous metaplasia in 5 cases.

The molecular analysis of pulmonary tissues collected from every autopsy revealed a high positivity rate for all three SARS-CoV-2 genes in 14 out of 15 cases. One case showed positivity for two out of three genes, namely the *N* and *ORF1ab* genes. Immunohistochemistry also demonstrated a high positivity count in lung tissues, with the nucleocapsid protein detected in 12 out of 15 cases, predominantly in type II pneumocytes and multinucleated giant cell-like aggregates (all 12 cases), alveolar macrophages (9 cases), or fibroblasts (6 cases), as well as in hyaline membranes in cases where such formations were observed (5 cases) [7].

### 2.2. Heart Tissue Analysis

**Figure 2:**
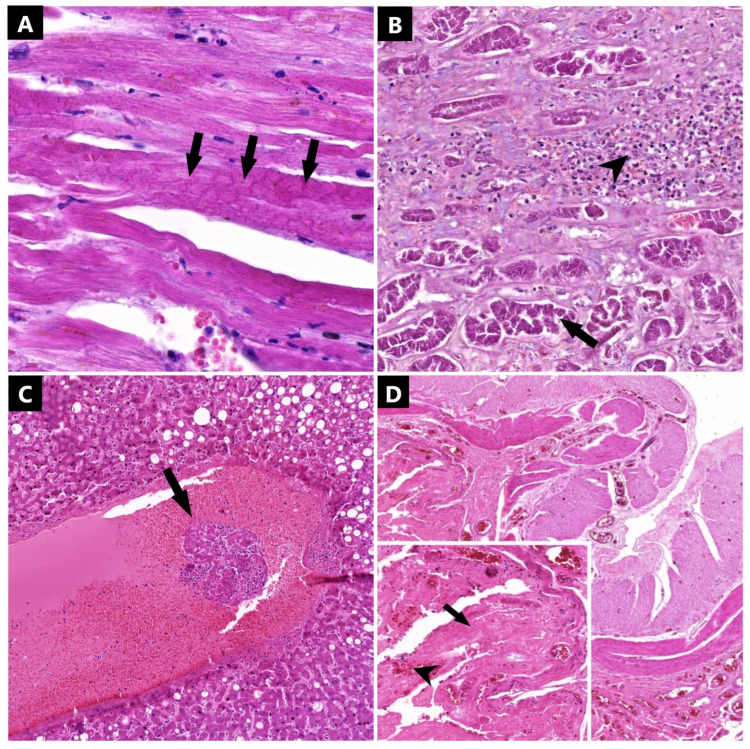
Microscopic aspect of tissue samples collected (hemotoxylin-eosin (HE)-staining): (**A**) myocardium (1050×)—contraction band necrosis (arrows); (**B**) renal parenchyma (328×)—acute tubular necrosis (arrows) with scattered lymphocytes around (arrowhead); (**C**) liver (119×)—vascular thrombosis (arrow); and (**D**) small intestine (105×) with insert (490×)—diffuse mucosal erosion with mucosal hemorrhage (arrowhead) and necrosis (arrow).

After histopathological analysis of the myocardium, we observed microstructural myocytic injuries in 12 cases represented by undulation or the fragmentation of the myocardial fibers, nuclear pyknosis, or vacuolar myocytic degeneration. We found injuries related to myocardial necrosis to be highlighted by the presence of contraction bands in two cases (Figure 2). Myocytic suffering was found in six cases, where we observed cardiomyocytes with large, angular, or irregular nuclei with central nucleoli, irregular chromatin patterns, or binucleation. Regarding inflammatory changes, no myocarditis was observed; only two cases presented inflammation at the epicardial and subepicardial levels, which was mainly formed by groups of lymphocytes, and three cases presented large amounts of neutrophils in the vascular lumen of the myocardium. Thirteen out of fifteen cases presented chronic injuries translated by myocardofibrosis (eleven cases), hypertensive cardiomyopathy (seven cases), hypertrophic cardiomyopathy (four cases), or cardiac lipomatosis (four cases).

Following rt-qPCR analysis of the heart tissue, we concluded that 7 out of 15 cases studied presented positivity for all three viral genes; in two cases, we detected only two out of three genes, and in two cases, we managed to detect only one out of three genes (Table 1). From the study group in which the detection of all three viral genes was possible, only one presented contraction band necrosis, four of them presented myocytic cytopathic effects, and in two patients, the viral nucleocapsid was detected. Out of the three cases that presented neutrophils within the vascular lumen, two of them also presented the SARS-CoV viral genome, but none of them were positive for the SARS-CoV-2 nucleocapsid antibody.

**Figure 3:**
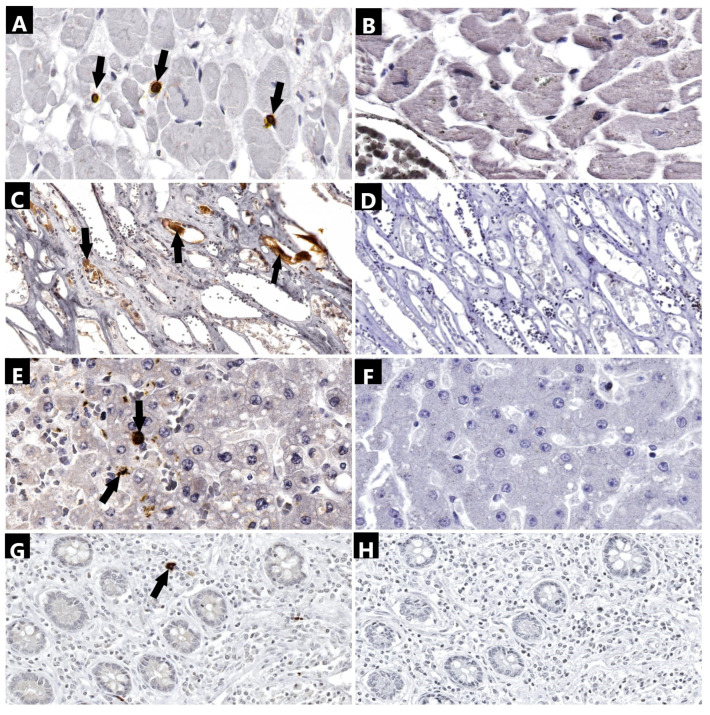
Immunohistochemical analysis of tissue samples collected (anti-SARS-CoV-2 nucleocapsid antibody): (**A**) myocardium (2240×)—positivity in interstitial macrophages (arrows); (**B**) myocardium (2240×)—negative control; (**C**) renal parenchyma (600×)—positivity in injured tubular cells (arrows); (**D**) renal parenchyma (600×)—negative control (**E**) liver (1730×)—positivity in hepatocytes and Kuppfer cells (arrows); (**F**) liver (1730×)—negative control; (**G**) small intestine (590×)—positivity in lamina propria macrophages (arrows); and (**H**) small intestine (590×)—negative control.

SARS-CoV-2 IHC demonstrated the presence of nucleocapsid in the cardiac tissue only in three cases (Table 2), mainly in the fibroblasts and cardiac interstitial macrophages, with no detection at the level of the myocytes (Figure 3). From these three cases, two patients were also positive for all three viral genes after molecular analysis; two of them presented with myocytic cytopathic effects on histopathology and only one patient presented with lymphocytic pericarditis. No patient suffering myocardial necrosis presented active viral particles within the cardiac parenchyma.

### 2.3. Kidney Tissue Analysis

Regarding the kidneys, in 5 cases, we observed injuries consistent with acute tubular necrosis (Figure 2); 2 cases presented with a mild vasculitic reaction but with no clear changes related to glomerulonephritis or microthrombosis. Also, in seven cases, we observed marked vascular congestion in the blood vessels at the medulla level. Inflammation was found in 11 out of 15 cases, represented by a lymphocytic infiltrate. Chronic injuries were constantly found at this level, with 12 cases presenting with interstitial sclerosis, 7 cases presenting with glomerular sclerosis, and 4 cases presenting with vascular sclerosis. In two cases, we detected multiple benign retention cysts, and in one case, we found a metastasis of epithelial origin.

All three SARS-CoV-2 genes were present at this level in 6 cases, as well as 4 cases in which we detected only two genes and 3 cases positive for only one gene (Table 1).

The nucleocapsid protein was found only in two cases (Table 2) with weakly positive staining at the level of the tubular cells or hyaline casts, but most importantly, both patients presented with acute tubular necrosis on histopathology (Figure 3). Another two patients presented with lesions consistent with acute tubular necrosis without the detection of the viral nucleocapsid but with the detection of the viral genome, and the remaining two patients presented with acute tubular lesions without viral interventions.

### 2.4. Liver Tissue Analysis

Tissue samples collected from the liver were studied using histopathological techniques, revealing acute liver injuries in 13 out of 15 patients. Among them, 4 exhibited granulo-vacuolar hepatocytic dystrophy, 2 presented with neutrophil vascular mobilization, and a significant number (10 cases) showed inflammation of the portal tracts, mainly mediated by lymphocytes. Additionally, five samples displayed hepatocytes with large, slightly irregular nuclei, rough, inhomogeneous chromatin, single or multiple visible nucleoli, and occasional binucleation, indicating acute hepatocytic suffering. No microscopic signs of vasculitis were observed, and only three cases displayed a few blood vessels containing microthrombi in the lumen (Figure 2). Chronic liver injuries were found in 13 patients, with 11 suffering from hepatic steatosis, 6 presenting with alterations consistent with chronic passive congestion, and 6 showing portal or periportal fibrosis.

Among the patients, all three viral genes were detected in the livers of 7 patients, while 3 patients tested positive for only two viral genes (Table 1).

IHC analysis revealed positivity in the hepatocytes of 7 patients (Figure 3), with 1 patient showing only weak positive staining for the nucleocapsid antibody. Antibody binding was observed at the level of the Kupffer cells in 3 patients and at the level of interstitial fibroblasts in 2 patients (Table 2). Among the 10 samples with a periportal lymphocytic inflammatory infiltrate, the viral nucleocapsid was detected in 7 of them at the level of the hepatocytes, fibroblasts, or Kupffer cells. Additionally, in 4 out of 5 patients with cytopathic effects at the hepatocyte level, the SARS-CoV-2 nucleocapsid and the *N*, *S*, and *ORF1ab* genes were detected. Similar findings were observed in 4 cases with lesions consistent with acute granulo-vacuolar dystrophy, where 3 out of 4 cases tested positive for the nucleocapsid and the three viral genes. Vascular microthrombi were associated with the presence of the nucleocapsid and viral genome only in one case. The presence of neutrophils within the vascular lumen was not associated with the presence of SARS-CoV-2 within the liver parenchyma.

### 2.5. Spleen Tissue Analysis

The spleen exhibited unspecific changes mainly at the level of the white pulp, with 8 samples showing white pulp atrophy, 5 samples displaying relatively normal lymphoid follicles but lacking germinal centers, and 2 revealing lymphoid hyperplasia.

Among the spleen samples subjected to rt-qPCR analysis, 6 showed positivity for all three genes, 5 showed positivity for two genes, and 2 showed positivity for only one out of three genes (Table 1).

Intense positivity for the SARS-CoV-2 nucleocapsid antibody was observed at the level of splenic macrophages in nine cases. Among the 8 cases presenting white pulp atrophy, 5 also showed the presence of the nucleocapsid at the level of the macrophages, but only 3 cases displayed the SARS-CoV-2 viral genome (Table 2).

### 2.6. Intestinal Tissue Analysis

The histopathological evaluation of the intestines revealed unspecific changes in all collected samples, such as the lympho-monocytic and/or macrophagic inflammatory infiltrate in nine cases. Only two cases showed an inflammatory infiltrate composed of neutrophil clusters at the level of the colon. Erosions were observed in 7 cases, necrosis in 2 cases, and hemorrhage in 3 cases at the mucosal level of the small intestine. Similarly, colon samples showed erosions in 7 cases, necrosis in 2 cases, and hemorrhage in 3 cases (Figure 2). Additionally, both the colon and small intestine exhibited chronic regenerative injuries consistent with mucosal and/or submucosal fibrosis in 6 patients for the small intestines and 4 patients for the colon. Glandular atrophy was observed in the colons of three patients.

Tissue samples from the small intestines tested positive for the *N*, *S*, and *Orf1ab* genes in 7 patients, while these three genes were detected in the colon samples of 8 patients. Two out of three genes were detected in 3 and 4 cases, respectively. Only one single gene was detected in two small intestine samples (Table 1).

Microscopic analysis using the SARS-CoV-2 nucleocapsid antibody revealed the presence of the nucleocapsid protein in the macrophages of the lamina propria in both the colon and small intestine of 12 patients (Figure 3; Table 2). There was no correlation found between mucosal necrosis or hemorrhage and the presence of the viral nucleocapsid or the SARS-CoV-2 genome at this level.

## 3. Discussion

The histopathological analysis at the cardiac level revealed a range of myocardial inflammatory and associated chronic lesions within our study cohort. The association between the myocardial inflammatory infiltrate and evident microscopic lesions at the level of cardiomyocytes, particularly myocardial injury, is representative of active lymphocytic myocarditis according to the Dallas classification. The presence of an interstitial lymphocytic infiltrate without evident myocardial lesions determines the so-called borderline myocarditis, according to the same classification [8]. There was no evidence of microthromboses, myocarditis, or vasculitic reaction in our study cohort. Leukocytoclastic vasculitis is defined by the presence of fibrinoid necrosis, perivascular hemorrhage, and massive infiltration of the vascular wall with numerous neutrophils [9]. In a limited number of cases, we observed the presence of a lymphocytic inflammatory infiltrate at the level of the pericardium and within the subepicardial space. Additionally, it is known that viral infections can trigger a pericardial immune reaction, which is usually self-limited, transient, and termed acute benign pericarditis [10]. Nonspecific ischemic lesions, which do not necessarily have a direct causative relationship with viral infection, were detected in the majority of patients in the study group. On the other hand, lesions with a much higher specificity for a viral infection, such as vasculitic reaction or myocarditis, were placed in the opposite plane, with no cases from our study cohort presenting these types of lesions. This indicates that the affinity of the SARS-CoV-2 virus for cardiac tissue is low, confirmed by the molecular and immunohistochemical analyses of postmortem specimens collected at this level. Chronic injuries were in the form of myocardofibrosis, hypertensive cardiomyopathy, hypertrophic cardiomyopathy, or cardiac lipomatosis. Myocardial fibrosis represents the accumulation of collagen in the myocardial tissue, either through diffuse fibrous tissue deposition within the interstitium, resulting in the secondary thickening of the extracellular matrix, or through collagen accumulation around the intramyocardial blood vessels, or by replacing myocardial fibers destroyed by dense fibroconnective tissue. These lesions generate the following three spectra of myocardial fibrosis: interstitial myocardial fibrosis, perivascular myocardial fibrosis, and replacement myocardial fibrosis [11]. In hypertrophic cardiomyopathy, the microscopic appearance of the myocardium was characterized by the presence of elongated myocardial fibers with irregular arrangement and varied shapes, along with myocytes exhibiting large, bizarre nuclei, often polygonal in shape, some of which were binucleated and frequently associated with myocardial fibrosis [12].

In the case of the heart, the detection of the *N*, *S*, and *ORF1ab* genes, as well as the viral nucleocapsid, was established in only a limited number of cases. Linder et al. conducted 39 autopsies on patients who died secondary to SARS-CoV-2 infection and stated that there were no differences in inflammatory infiltrates between patients with documented cardiac infection and those without cardiac infection. The authors claimed that the SARS-CoV-2 virus is not found in cardiomyocytes but predominantly in interstitial cells or macrophages present in the myocardium, similar to our study [13]. Examining 16 cases of deaths secondary to SARS-CoV-2 infection, Kawakami et al. identified the presence of the SARS-CoV-2 virus in the myocardium in only two cases [14].

The predominant histopathological changes at the renal level were primarily chronic in nature. The interstitial lymphomonocytic inflammatory infiltrate constituted the largest proportion of acute lesions, followed by vascular medullary congestion in the renal medulla, acute tubular necrosis, and, in a limited number of cases, a lymphocytic vasculitic reaction in the walls of small blood vessels. Marked medullary congestion is a nonspecific alteration frequently observed during autopsy in cases of prolonged agony, often secondary to heart failure, resulting in decreased cardiac output, increased intra-abdominal pressure, or elevated venous pressure [15]. After a thorough review of the specialized literature, we noticed the same histologic pattern described in our study. However, some authors have demonstrated a higher incidence of acute lesions, particularly those of the renal tubules. Rivero J. et al. analyzed renal tissue specimens collected from 85 patients and observed that acute tubular lesions could be detected in 84% of cases but without glomerular lesions [16]. A meta-analysis conducted on a sample of 954 patients concluded that the incidence of acute tubular lesions in COVID-19 patients is 85%, renal vascular congestion is observed in 66% of patients, followed by the presence of interstitial inflammatory infiltrate in 27% of cases, and the formation of microthrombi in 12% of cases. Less frequent lesions were observed in 7% of cases for endothelial lesions [17]. Similar lesions were described by Torge D. et al. in a review published in 2022 [18].

We determined, using the rt-qPCR technique on renal tissue samples, that a total of 6 out of 15 cases tested positive for the *S*, *N*, and *ORF1ab* genes. The presence of these genes was observed in only 40% of patients, and the viral nucleocapsid was detected in only 13% of patients, all of whom presented with acute tubular necrosis on histopathology. The pathological mechanism of acute tubular necrosis in SARS-CoV-2 can be multifactorial, involving active viral invasion, marked viremia, hypoxia, or an exaggerated systemic immune response. Acute tubular necrosis can also be caused by alterations in the microvascularization of the nephron during hemodynamic disturbances or hypoxic nephropathy in critically ill patients [19]. One study confirmed the presence of the viral genome in 50% of cases and the nucleocapsid in 31% of cases [17], indicating a higher incidence compared to our study. On the other hand, Massoth L. et al. analyzed multiple specimens, including those from the renal level, but this time from living patients, and the result was negative for extrapulmonary organs in rt-PCR, immunohistochemistry, and in situ hybridization analyses [20]. Other authors also noticed these discrepancies, raising controversies regarding the virus’s affinity for renal parenchyma. To demonstrate this aspect, multiple detection techniques would be needed, including rt-PCR, IHC, in situ hybridization (ISH), electron microscopy, and immunoelectron detection [21]. A study from China, published in 2021, described the renal injury mechanisms in patients with SARS-CoV-2 infection. The authors describe the following two main mechanisms: direct viral injury, generated by the binding of the spike protein to ACE2 receptors, which is mostly expressed in the epithelium of proximal tubules and podocytes, as well as a second mechanism of immunological mediation. Following the formation of the spike protein–receptor complex, the latter becomes less expressed on the cell surface, leading to an accumulation of angiotensin II, which enhances both local and systemic inflammation, causing tubular injuries, primarily in the proximal tubules and minimal glomerular injuries in podocytes. The second mechanism is the indirect one, mediated by systemic immune mechanisms, endothelial injuries, thrombus formation, and hypoxia [22].

Following a hepatic histopathological examination, we observed that the highest proportion of microscopic lesions was represented by associated chronic lesions, followed by hepatocellular necroinflammatory lesions like granulo-vacuolar dystrophy or lympho-monocytic periportal inflammatory infiltrate, and in a limited number, vascular lesions like microthrombosis. Hepatocellular dystrophy is itself nonspecific and is observed in both acute infectious hepatobiliary conditions and non-infectious hepatitis, and can also be seen in chronic liver conditions, for example, in hepatic dystrophies associated with metabolic syndromes [23]. Periportal inflammation can be triggered by a wide range of conditions, including some with necro-inflammatory activity, including viral hepatitis, autoimmune hepatitis, or drug-induced hepatitis. Additionally, inflammatory infiltration in the portal space can be observed in certain cholestatic diseases, such as primary biliary cholangitis, primary sclerosing cholangitis, or metabolic diseases [24]. The same pattern of hepatocellular lesions has been described by other authors in the specialized literature [18,25]. A recently published study succinctly described the pathogenic mechanisms of hepatocellular lesions in patients infected with the SARS-CoV-2 virus. Thus, hepatocellular lesions can arise due to direct viral infection at the hepatocellular level are mediated by the systemic inflammatory response, especially in patients diagnosed with cytokine storm where high concentrations of IL6 and IL1 generate acute hepatocellular injuries, arise due to hypoxia or are caused injuries secondary to drug treatment within the anti-COVID-19 therapeutic protocol [26]. On another note, Pesti A. et al. studied 20 autopsy cases and analyzed hepatic tissue using molecular techniques, such as IHC and ISH. From a total of 20 patients, the viral genome could be detected in 13 of them by rt-qPCR, and nucleocapsid was detected in 12 patients at IHC in fibroblasts, macrophages, and also in endothelial cells, in contrast to our study [27].

The histopathological changes in the splenic parenchyma mainly involved disorganization at the level of the white pulp. Most of the anomalies detected at the microscopic level included a variable decrease in white pulp volume, ranging from mild hypoplasia, where a slight decrease in the number of lymphoid follicles was observed, with discrete effacement of the mantle zone and the loss of the boundary between the white pulp and the red pulp, to atrophy, represented by severe structural disorganization of the white pulp, with the loss of the follicular pattern and a drastic decrease in the number of lymphocytes, both at the follicular and parafollicular levels. These changes can be induced even in an infectious context [28,29]. The presence of the viral genome and nucleocapsid at the splenic level is evident due to the blood-filtering function of this organ. However, the histopathological changes did not appear necessary as a direct effect of the virus on the splenic parenchyma, as shown by our results. The disorganization of the white pulp, which is related to the lymphocytopenic effect of the viral infection on hematopoietic organs, may also be the consequence of the direct viral invasion of T lymphocytes, or rather by the innate immunity dysregulation with the constant release of proinflammatory cytokines, like Il-6 or Il-1, including from infected spleen macrophages, which, in turn, generate lymphocytic apoptosis [30].

While this study provides valuable insights, it is important to acknowledge several limitations. Firstly, our inability to determine the exact SARS-CoV-2 variant due to the absence of a sequencer in our laboratory limited our ability to comprehensively characterize the viral strains involved in the observed cases. Moreover, the IHC findings lacked validation through ISH, which is a technique that could have offered deeper insights into the specific cell and tissue types susceptible to invasion by the novel coronavirus, thus enhancing the precision of our analyses. Additionally, the analysis was constrained by the relatively small number of cases examined, which may limit the generalizability of our findings. Furthermore, the absence of an examination of brain tissue in our study is notable, as a large number of autopsies were performed only at the level of the thoraco-abdomino-pelvic cavities, leaving the cranial cavity intact. However, we recognize the importance of studying the influence of the SARS-CoV-2 virus on cerebral tissue, and we plan to address this in future research after increasing the specimen number and incorporating brain tissue analysis. Despite these limitations, our research contributes significantly to understanding the pathophysiological mechanisms underlying organ-specific injury in COVID-19, laying the groundwork for further investigation.

## 4. Materials and Methods

We examined 15 hospitalized patients infected with the SARS-CoV-2 virus between March and October 2021 who eventually succumbed to severe complications due to the infection at the Sibiu County Clinical Emergency Hospital of Sibiu, Romania. During this timeframe, the dominant variant was *Alpha* (*Lineage B.1.1.7*), initially followed by its replacement by *Delta* (*Lineage B.1.617.2*). All patients were ethnic Romanians.

Autopsies were conducted in a COVID-19-restricted area, following national protocols and safety measures. We conducted detailed macroscopic examinations and collected tissue samples from multiple organs, including the myocardium, kidneys, liver, spleen, small and large intestines, for histopathological examination to study the morphopathological changes and rt-qPCR analysis to detect the SARS-CoV-2 viral genome. Additionally, immunohistochemical analysis was performed to detect the viral nucleocapsid. The autopsies were conducted between 6 and 24 h after death, and tissue samples were collected within this timeframe.

For the preparation of histopathological slides, tissue samples obtained from each autopsy underwent chemical processes of fixation, between 24 h and no longer than 72 h, dehydration, and processing, as well as mechanical processes of embedding and microtome sectioning and subsequent staining processes. Before actual processing, following an initial fixation of the tissue material in 10% formaldehyde for at least 48 h, the collected tissues were sectioned to a thickness of about 2–3 mm, shaped, and macroscopically oriented, then embedded in classic histological cassettes with lids. The histopathological processing of the shaped and macroscopically oriented material in histological cassettes was performed using a rotary histoprocessor, Epredia Revos (Epredia, Kalamazoo, MI, USA). After tissue materials were processed, they were embedded in paraffin blocks for subsequent microtome sectioning. Sections were cut using a manual microtome at 2 or 3 μm, depending on the tissue, with a vertical stroke of 20 mm; these were then stretched with the help of a warm water bath and laid on positively charged slides. After completing the deparaffinization and rehydration baths, the slides were rinsed with tap water and transferred to the staining compartment. All histopathological slides in the current study were stained only with the standard Hematoxylin-Eosin stain using an automated histological stainer. After staining, dehydration, and final clarification were completed, the slides were air-dried and then mounted using Canada balsam. The process is similar to other works of research conducted by our team [31].

**Figure 4:**
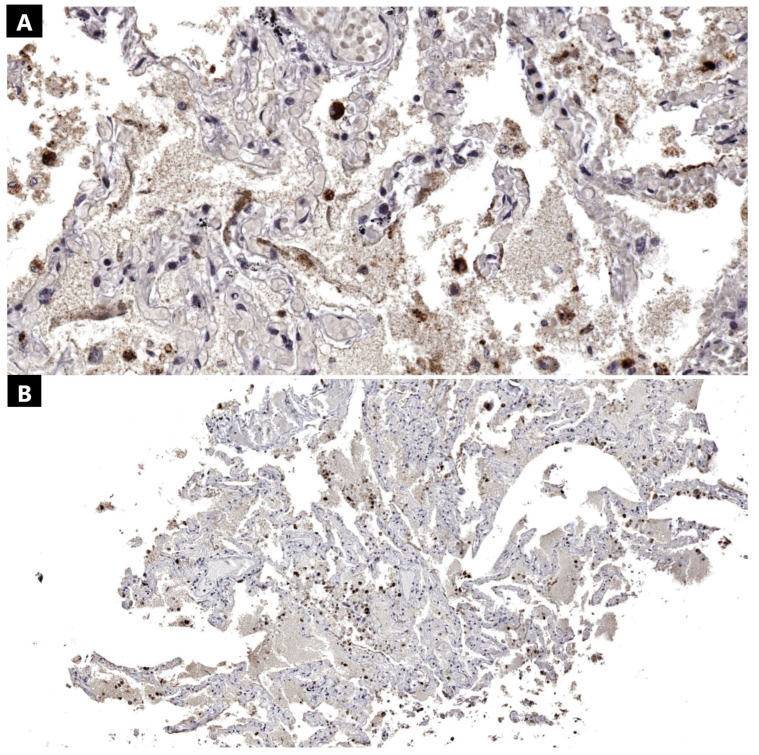
Lung tissue positive control (Anti-SARS-CoV-2 nucleocapsid antibody): (**A**) Lung (870×); (**B**) lung (210×).

**Figure 5:**
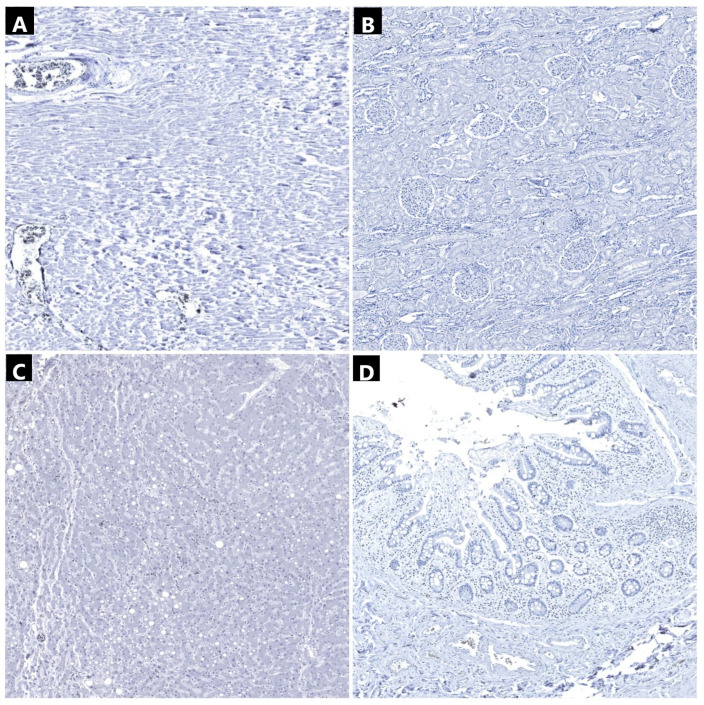
Immunohistochemical analysis of tissue samples collected (anti-SARS-CoV-2 nucleocapsid antibody): (**A**) myocardium (120×)—negative control; (**B**) kidney (110×)—negative control; (**C**) liver (170×)—negative control; and (**D**) intestine (210×)—negative control.

The technique for antibody incubation was automated, utilizing the Epredia Autostainer 360 (Epredia, Kalamazoo, MI, USA). Tissue specimens embedded in paraffin wax were manually sliced using a microtome to a thickness of 3–5 microns. Following this, these sections were fixed to positively charged microscopic slides to ensure secure adhesion. The slides containing the sliced tissue specimens were left to incubate for 2 h at 58 °C. Prior to epitope demasking, the slides underwent deparaffinization with xylene and rehydration using ethanol at decreasing concentrations. Epitope recovery was facilitated using the ImmunoDNA Retriever kit (BioSB, Santa Barbara, CA, USA) with a citrate buffer solution at a pH level of 6. The primary antibodies utilized were sourced from the IgG mouse monoclonal isotype. This set included the SARS-CoV-2 nucleocapsid antibody for identifying the viral nucleocapsid within the cytoplasm of infected cells. The antibody was pre-mixed, negating the need for a dilution protocol. For the chromogenic immunohistochemical reaction, we utilized an anti-mouse HRP IgG secondary antibody with the DAB chromogen. Hematoxylin served as the counterstain. The immunohistochemical preparation adhered to the same protocol as previous research conducted in our laboratory [32]. For the positive controls, we acquired Hydrophilic Plus slides containing SARS-CoV-2 positive lung tissues (Figure 4). To establish a negative control for the primary antibody, we utilized a nonspecific mouse monoclonal IgG isotype for all SARS-CoV-2 positive immunohistochemical sections examined (Figure 3). Additionally, for negative controls, tissue specimens obtained from three pre-pandemic patients who died from a massive cerebral hemorrhage, ischemic stroke, and aortic rupture were analyzed (Figure 5).

All microscopic slides were digitalized using the Panoramic Desk II DW digital slide scanner (3DHistech, Budapest, Hungary) and examined with the 3D Histech Slide Viewer application.

For genetic analysis, tissue samples were immediately preserved in an RNA lock reagent upon collection and stored at −20 °C for no longer than 20 days, depending on the number of samples collected at that time. The ARN was extracted with the help of the QIAamp viral RNA extraction kit (Qiagen, Hilden, Germany) following the manufacturer’s instructions. To validate the RNA extraction process and ensure no inhibitors were present in the RT-qPCR reactions, in vitro-generated *MS2* RNA was added to the sample lysis buffer as a control. The presence and quantity of the *N* (*nucleocapsid*), *S* (*spike*), and *Orf1ab* SARS-CoV-2 viral genes, along with the *MS2* control, were determined via reverse-transcription quantitative PCR using the TaqPath COVID-19 CE-IVD RT-PCR kit (Thermo-Fisher Scientific, Waltham, MA, USA). Samples were considered positive for N, S, or Orf1ab gene targets if the threshold cycle (Ct) was below 40 cycles [7].

## 5. Conclusions

The most frequent acute microscopic lesions in the cardiac tissue were microstructural changes in myocardial fibers, followed by cytopathic changes in cardiomyocytes, as well as myocardial necrosis or lymphocytic pericarditis. Lymphocytic myocarditis and microvascular thrombi were not observed in the specimens examined from the current study cohort. The presence of neutrophils in the myocardial vascular lumen was not associated with viral nucleocapsid presence at this level; however, it was associated in two out of three cases with viral genome detection, indicating that the neutrophil-mediated immune response is rather a consequence of marked viremia. Viral nucleocapsid detection in cardiac fibroblasts and/or macrophages was associated in 2 out of 3 cases with viral genome detection by PCR and also in 2 out of 3 cases with myocardial injury, and in 1 case with lymphocytic pericarditis.

The most common renal alteration was the presence of a lympho-monocytic inflammatory infiltrate at the interstitial level. Acute tubular necrosis in patients with SARS-CoV-2 infection may be triggered by active virus presence in tubular cells but is rather a consequence of systemic hypoxia, vascular lesions, or an acute aberrant inflammatory response and is more commonly associated with increased viremia. The inflammatory response in the renal parenchyma is mediated by the presence of an interstitial lympho-monocytic inflammatory infiltrate and may be linked to the presence of the virus at this level.

The predominant hepatic histopathological alteration observed in COVID-19 patients is the presence of a lympho-monocytic inflammatory infiltrate in the portal space, followed by the cytopathic effects of hepatocytes and hepatocellular granulo-vacuolar dystrophies. These three lesions were primarily associated with the presence of the viral nucleocapsid in the hepatic parenchyma, although to a lesser extent, they were also a consequence of an exaggerated inflammatory response in these patients.

Splenic alterations are consequences of red pulp disorganization translated by marked congestion at this level, followed by white pulp disorganization, most commonly secondary to follicular atrophy, which has a direct relationship with the long-term evolution of the infection and is likely a consequence of the systemic lymphopenia observed in these patients.

The histopathological changes in the intestines were nonspecific, primarily characterized by a lympho-monocytic inflammatory infiltrate at the mucosal level, accompanied by epithelial erosions and mucosal fibrosis. Intestinal mucosal necrosis and hemorrhage were observed in a limited number of cases, with no apparent correlation with the presence of viral nucleocapsid or genome at this level.

The *N*, *S*, and *ORF1ab* genes showed a detection frequency by rt-qPCR in intestinal, splenic, hepatic, cardiac, and renal tissues, which was approximately similar, with a maximum of 8 positive cases in intestinal tissue and a minimum of 6 positive cases in splenic tissue for all three viral genes studied. The detection of the SARS-CoV-2 viral nucleocapsid was most frequently observed in intestinal tissue (12 cases), followed by splenic, hepatic, cardiac, and renal tissue, with the latter exhibiting only two positive cases. Following the correlation between the results of rt-qPCR and IHC, it was concluded that the SARS-CoV-2 virus is most frequently present and replicative in intestinal, hepatic, and splenic tissues. The most significant disparity between the detection of the viral genome and the viral nucleocapsid was noted in cardiac and renal tissues, suggesting a low affinity of the virus for these two organs. It was observed that most patients with viral genome detection at this level were in a viremic phase. The cells directly targeted by the SARS-CoV-2 virus are hepatocytes, renal tubular cells, interstitial fibroblasts from the myocardium, hepatocytic fibroblasts, and Kupffer cells, as well as splenic and intestinal macrophages.

## Figures and Tables

**Table 1:** RT-qPCR results of the study group.

	Cycle Threshold			
	*MS2*	*N* Gene	*ORF1ab*	*S* Gene
Case 1 Lung	32.651	28.140	27.811	28.061
Case 1 Heart	32.651	28.140	27.811	28.061
Case 1 Kidney	27.217	29.937	29.514	30.842
Case 1 Liver	30.272	22.867	25.088	25.481
Case 1 Spleen	28.788	23.045	25.138	25.591
Case 1 Small int	27.185	25.608	27.093	27.776
Case 1 Colon	24.317	29.847	30.777	31.744
Case 2 Lung	33.087	17.991	19.012	19.247
Case 2 Heart	28.711	19.096	19.298	19.989
Case 2 Kidney	28.849	21.676	22.589	22.638
Case 2 Liver	28.951	23.128	23.950	23.855
Case 2 Spleen	28.182	23.919	25.000	25.013
Case 2 Small int	26.410	23.770	24.243	24.314
Case 2 Colon	35.281	23.793	24.980	25.292
Case 3 Lung	31.592	15.776	16.532	16.699
Case 3 Heart	34.721	26.321	28.074	29.312
Case 3 Kidney	29.318	23.375	24.437	24.845
Case 3 Liver	39.000	37.170	35.259	36.383
Case 3 Spleen	27.161	26.698	26.636	26.808
Case 3 Small int	29.914	27.651	26.403	26.221
Case 3 Colon	31.445	23.566	24.227	24.183
Case 4 Lung	29.302	23.325	24.256	24.436
Case 4 Heart	27.183	28.258	30.105	30.272
Case 4 Kidney	26.865	31.353	32.459	33.419
Case 4 Liver	26.422	26.502	27.406	26.958
Case 4 Spleen	27.937	32.612	32.766	32.923
Case 4 Small int	31.320	34.093	34.421	33.804
Case 4 Colon	28.718	32.263	32.451	32.345
Case 5 Lung	29.047	34.308	35.055	34.756
Case 5 Heart	26.405	Ø	Ø	Ø
Case 5 Kidney	26.221	Ø	Ø	Ø
Case 5 Liver	25.486	Ø	Ø	Ø
Case 5 Spleen	27.472	Ø	Ø	Ø
Case 5 Small int	25.896	Ø	Ø	Ø
Case 5 Colon	25.475	33.207	33.033	33.950
Case 6 Lung	33.122	21.819	19.108	25.586
Case 6 Heart	27.628	34.247	30.799	37.939
Case 6 Kidney	27.913	30.697	33.570	Ø
Case 6 Liver	27.700	34.555	33.469	Ø
Case 6 Spleen	26.521	34.077	32.863	Ø
Case 6 Small int	27.392	Ø	24.159	Ø
Case 6 Colon	30.223	31.587	31.274	33.926
Case 7 Lung	28.718	22.249	18.069	28.168
Case 7 Heart	27.084	Ø	Ø	Ø
Case 7 Kidney	27.111	37.829	Ø	Ø
Case 7 Liver	29.466	Ø	Ø	Ø
Case 7 Spleen	28.454	16.743	15.159	Ø
Case 7 Small int	27.488	32.224	15.659	Ø
Case 7 Colon	27.990	33.896	24.764	Ø
Case 8 Lung	27.191	29.079	29.185	35.269
Case 8 Heart	27.049	Ø	11.259	Ø
Case 8 Kidney	24.421	19.596	20.489	19.995
Case 8 Liver	28.034	Ø	Ø	Ø
Case 8 Spleen	26.340	Ø	38.556	Ø
Case 8 Small int	25.342	Ø	Ø	Ø
Case 8 Colon	26.856	Ø	Ø	Ø
Case 9 Lung	28.711	19.096	19.298	19.989
Case 9 Heart	28.417	Ø	Ø	Ø
Case 9 Kidney	26.047	30.424	19.897	Ø
Case 9 Liver	27.092	30.947	32.216	Ø
Case 9 Spleen	27.425	32.168	32.438	Ø
Case 9 Small int	30.044	Ø	Ø	Ø
Case 9 Colon	31.691	Ø	Ø	Ø
Case 10 Lung	28.847	29.571	30.486	23.025
Case 10 Heart	25.702	32.637	33.713	Ø
Case 10 Kidney	28.210	33.545	34.509	Ø
Case 10 Liver	26.534	32.126	32.666	20.317
Case 10 Spleen	26.239	33.209	33.245	Ø
Case 10 Small int	26.466	31.261	32.337	34.696
Case 10 Colon	27.445	31.375	32.035	Ø
Case 11 Lung	26.190	30.674	31.953	Ø
Case 11 Heart	26.335	23.367	23.411	Ø
Case 11 Kidney	26.882	28.915	29.475	Ø
Case 11 Liver	26.917	28.946	30.421	Ø
Case 11 Spleen	27.116	27.792	29.204	Ø
Case 11 Small int	26.939	27.545	27.910	Ø
Case 11 Colon	30.584	27.574	28.334	Ø
Case 12 Lung	24.731	30.403	24.160	32.997
Case 12 Heart	26.954	28.723	30.657	26.265
Case 12 Kidney	30.520	29.723	Ø	Ø
Case 12 Liver	27.676	31.092	33.505	33.989
Case 12 Spleen	26.591	28.189	28.845	28.982
Case 12 Small int	27.375	28.311	28.895	28.153
Case 12 Colon	26.497	29.722	30.776	31.197
Case 13 Lung	26.902	13.322	12.022	16.587
Case 13 Heart	26.405	Ø	Ø	Ø
Case 13 Kidney	26.221	Ø	Ø	Ø
Case 13 Liver	25.486	Ø	Ø	Ø
Case 13 Spleen	27.472	Ø	Ø	Ø
Case 13 Small int	25.475	33.207	Ø	Ø
Case 13 Colon	25.896	31.047	33.033	Ø
Case 14 Lung	26.429	33.384	32.192	35.215
Case 14 Heart	29.970	31.382	Ø	Ø
Case 14 Kidney	30.852	32.006	Ø	Ø
Case 14 Liver	27.371	Ø	Ø	Ø
Case 14 Spleen	27.128	29.87	Ø	Ø
Case 14 Small int	27.385	Ø	Ø	Ø
Case 14 Colon	28.220	Ø	Ø	Ø
Case 15 Lung	26.954	28.723	30.657	26.265
Case 15 Heart	26.665	28.214	29.038	29.349
Case 15 Kidney	27.095	22.040	22.686	22.814
Case 15 Liver	28.802	26.909	27.340	27.338
Case 15 Spleen	29.850	28.946	29.969	29.932
Case 15 Small int	28.739	27.183	27.873	28.162
Case 15 Colon	27.215	28.890	29.622	29.770

*MS2* = Emesvirus zinderi; *N* = nucleocapsid; *S* = spike; *ORF1ab* = open reading frame 1ab; Ø = No detection.

**Table 2:** Immunohistochemical results of the study group using the SARS-CoV-2 nucleocapsid antibody.

Case	Heart	Kidney	Liver	Spleen	Small Int	Colon
1	0	0	0	0	0	0
2	0	0	1	1	1	1
3	0	1	0	1	1	1
4	0	0	1	0	1	1
5	0	0	0	0	1	1
6	0	1	1	1	1	1
7	1	0	1	0	1	1
8	0	0	0	1	0	0
9	0	0	0	1	1	1
10	0	0	1	1	1	1
11	0	0	1	1	1	1
12	1	0	1	1	1	1
13	0	0	0	0	0	0
14	0	0	0	1	1	1
15	1	0	1	0	1	1
Total	3	2	8	9	12	12

1 = positive; 0 = negative.

## Data Availability

Data are contained within the article.
